# Mismatch-corrected CHIKV CDC Trioplex assay oligos restore sensitive pangenotype viral detection

**DOI:** 10.1128/jcm.00490-25

**Published:** 2025-06-11

**Authors:** Lika Aminata Diouf, Mignane Ndiaye, Diamilatou Balde, Agathe Shella Efire, Moussa Dia, Fatou Thiam, Manfred Weidmann, Oumar Faye, Idrissa Dieng

**Affiliations:** 1 Virology Department, Institut Pasteur de Dakar 89248, Dakar, Dakar Region, Senegal; 2 Ecole Supérieure Polytechnique, Université Cheikh Anta Diop de Dakar 89230 , Dakar, Dakar Region, Senegal; 3 Institute of Animal Hygiene and Veterinary Public Health University of Leipzig, Leipzig, Germany; 4 Institute of Microbiology & Virology, Brandenburg Medical School Theodor Fontane 477107 , Neuruppin, Brandenburg, Germany; 5 Animal Biology Department, Faculty of Sciences and Techniques, Cheikh Anta Diop University of Dakar 89230, Dakar, Dakar Region, Senegal; The University of North Carolina at Chapel Hill School of Medicine, Chapel Hill, North Carolina, USA

**Keywords:** chikungunya virus, CDC Trioplex RT-qPCR, underperformance, West African genotype, pan genotype, detection improvement

## LETTER

Chikungunya virus (CHIKV) is an arbovirus primarily transmitted by *Aedes* mosquitoes, predominantly in tropical and subtropical regions (1, 2). Due to global warming, the geographic spread of arboviruses, including CHIKV, is expanding, increasing their public health impact (3).

Genetically, CHIKV exists in three major genotypes: Asian, East-Central-South African (ECSA), and West African (WA) (1).

Although CHIKV originated in Africa, knowledge about its burden on the continent remains limited (2, 4). This is linked to low awareness, lack of effective surveillance programs, and reliable diagnostic tools (2).

Recently, Senegal experienced a significant CHIKV epidemic caused by the West African genotype, with up to 300 confirmed cases in late 2024 (5, 6). Additionally, a cluster of 12 imported cases belonging to this genotype was reported in France from Côte d’Ivoire in 2023 and 2024, respectively (4). These detections highlight an intensive and cryptic circulation of the virus in West Africa (4, 7).

To effectively monitor CHIKV spread in Africa and mitigate the risk of importation, reliable diagnostic tools are essential (4). For years, RT-qPCR has been the primary method for CHIKV detection (8). However, co-circulation of arboviruses such as Zika (ZIKV), Dengue (DENV), and CHIKV, which also share similar clinical pictures, poses diagnostic challenges (9). In response to this, the CDC Trioplex assay was developed for the simultaneous detection and differentiation of these viruses during the Zika outbreak in Latin America (9). Despite its broad utility, the Trioplex assay was reported to exhibit reduced sensitivity for the WA genotype, limiting its effectiveness in West Africa (6). To address this limitation, we developed AltoDesign, a novel RT-qPCR assay with optimized primers and probes designed to restore pangenotypic CHIKV detection (Table S1). This new oligonucleotide set was designed based on CHIKV sequences from GenBank, with a focus on recent Senegalese strains belonging to the WA genotype.

The AltoDesign assay’s ability to detect various CHIKV genotypes was evaluated in comparison to the in-house RT-qPCR assay and the CDC Trioplex assays using a panel of CHIKV strains available at the WHO Collaborating Center for Arboviruses at Institut Pasteur de Dakar (IPD) (Table S2). The performance of the AltoDesign, including the limit of detection (LOD), was assessed using a WA CHIKV strain (SH274640) with known viral titers in four technical replicates for probit analysis at 95% confidence.

Obtained standard curves from a 6-log serial dilution series showed that the assays exhibit amplification efficiencies of 110.17% and 92.35% for the CHIKV Trioplex and the AltoDesign, respectively, and that both assays show R^2^ values greater than 0.99.

**Fig 1:**
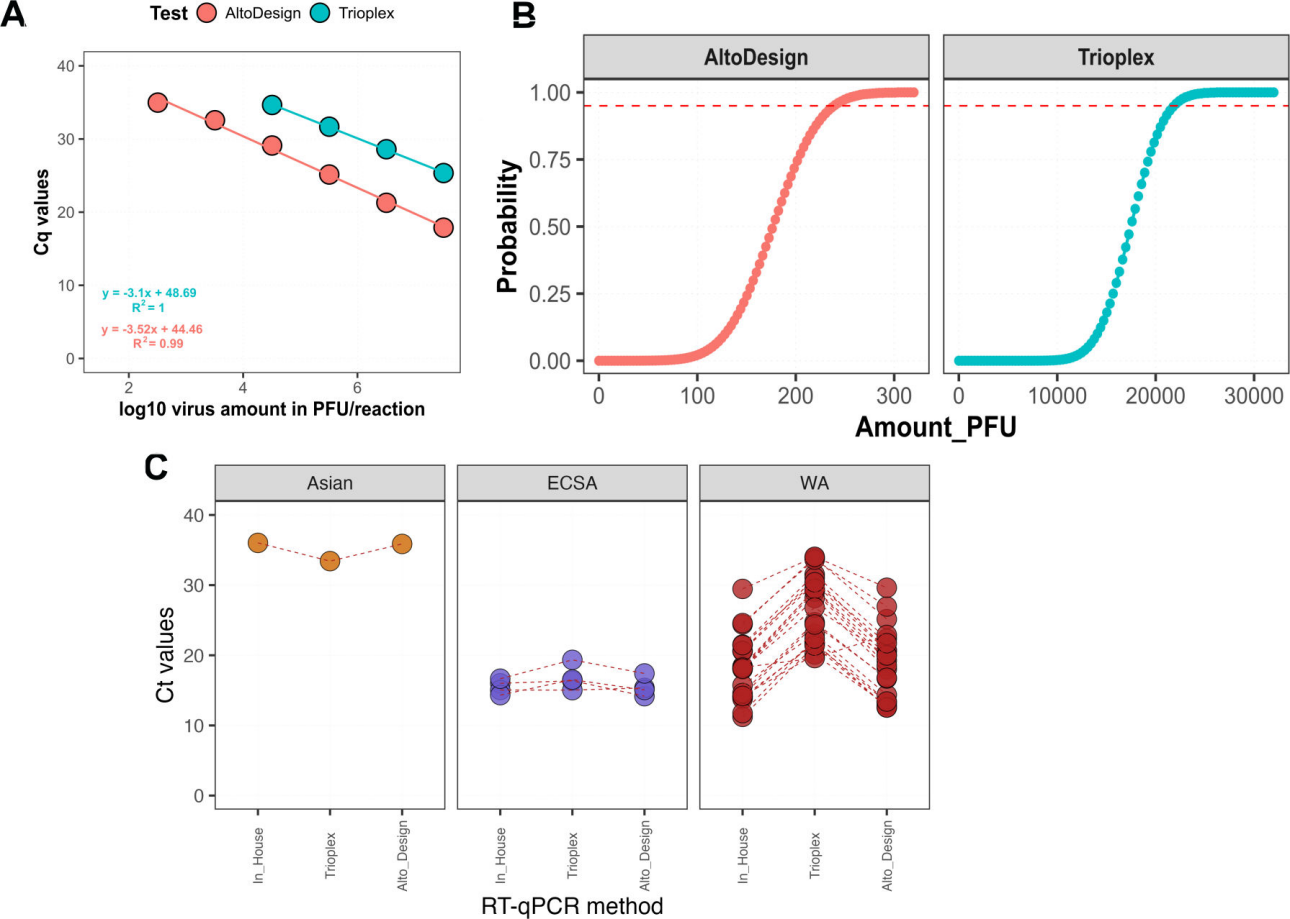
Comparative analysis of Trioplex, in-house, and AltoDesign assays performance. (**A**) Analytical sensitivity of Trioplex in comparison to AltoDesign on 10-fold serial dilutions of CHIKV WA strain with known viral titer. (**B**) Probit regression analysis of CHIKV RT-qPCR assays based on serial dilutions of CHIKV WA viral stock with known PFU titer. The probability of a positive result is plotted against the concentration of PFU per reaction. The 95% detection limit is indicated with the dashed lines. Probit values: 238.88 PFU/reaction for AltoDesign, and 21858.02 PFU/reaction for Triolplex. (**C**) Comparison of Cq values for CHIKV between IPD in house, CDC Trioplex, and AltoDesign across strains tested (Table S1)

The AltoDesign assays yield consistently lower Cq values (6.715 ± 0.88) than the CHIKV Trioplex assay, indicating increased efficiency and suggesting that mismatches described by Ndiaye and collaborators led to at least a 100-fold underestimation of the WA genotype template amount per reaction. Indeed, the limit of detection of the AltoDesign assay determined by using the PFU dilution series is about 5 Cq lower than the Trioplex (Fig. 1A), which roughly equates to an approximately 1–1.5 log₁₀ increased analytical sensitivity. In terms of PFU/reaction (log₁₀ 2), the AltoDesign assay detected 321 PFU/reaction (log₁₀ 2) with a mean Cq value of 34.97 ± 0.96, whereas the Trioplex assay had a detection limit of 32,100 PFU/reaction with a Cq value of 34.65 ± 0.36 (Table S3; Fig. 1A), which corresponds to an increased sensitivity of 2 log₁₀ steps. At 95% confidence, AltoDesign achieved a lower LOD (238.88 PFU/reaction) compared with the Trioplex assay (21858.02 PFU/reaction) (Fig. 1B). When testing CHIKV strain supernatants (Table S2), the AltoDesign for CHIKV WA genotype thus improves the lag Cq values previously described by Ndiaye and colleagues in comparison to the in-house assay and the Trioplex assay (Fig. 1C).

For RT-qPCR, previous work highlighted that mismatches on oligonucleotide binding sites can impair assay performances (10). Mismatches on target regions can lead to target failure, causing false negatives (11) or a drastic reduction in assay performances (6). In our study, we restored the sensitivity of the CHIKV CDC trioplex assay to efficiently detect the CHIKV WA genotype. The need for mismatch-corrected oligonucleotides to improve assay performance for emerging viral strains is a critical consideration in molecular diagnostics, particularly for pathogens like MPOX and influenza viruses, which exhibit high genetic variability (12, 13).

Additionally, the AltoDesign primers showed similar performance to both the IPD in-house assay (6) and the CDC Trioplex assay, with comparable Cq values observed across the tested samples of ECSA and Asian genotype strains (Fig. 1C; Table S2).

Our results show that the newly designed mismatches corrected oligonucleotides provide a more sensitive, accurate, and pangenotype detection of all available CHIKV genotypes.

The AltoDesign assay significantly improves CHIKV detection with a higher 95% limit of detection, particularly for the West African genotype, overcoming the limitations of the native CHIKV CDC Trioplex assay. Additionally, the updated oligos can still be combined with DENV and ZIKV oligos for differential viral identification while allowing pangenotype CHIKV detection with similar Cq values (Fig. S1). This optimized RT-qPCR system offers a more reliable diagnostic tool for enhanced surveillance and outbreak response in regions where CHIKV and/or flaviviruses sharing the same clinical presentation are endemic.

## SUPPLEMENTAL MATERIAL

Supplemental tables and figure jcm.00490-25-s0001.docx Tables S1 to S3 and Fig. 1.

ASM does not own the copyrights to Supplemental Material that may be linked to, or accessed through, an article. The authors have granted ASM a non-exclusive, world-wide license to publish the Supplemental Material files. Please contact the corresponding author directly for reuse.
