# Supplementary material for: Mismatch-corrected CHIKV CDC Trioplex assay oligos restore sensitive pangenotype viral detection
Source: J Clin Microbiol. 2025 Jun 11;63(7):e00490-25. doi: 10.1128/jcm.00490-25 (PMC12239721; doi:10.1128/jcm.00490-25)
Supplement: Supplemental tables and figure — Tables S1 to S3 and Fig. 1. [file jcm.00490-25-s0001.docx]

**Mismatche-corrected CHIKV CDC Trioplex assay oligos restore sensitive pang﻿genotype viral detection**

Lika Aminata Diouf^1$^, Mignane Ndiaye^1$^, Diamilatou Balde^1^, Agathe Shella Efire^1^, Moussa Dia^1^, Fatou Thiam^2^, Manfred Weidmann^3,4^, Oumar Faye^1^, Idrissa Dieng^1,5**^

^1^ Virology Department, Institut Pasteur de Dakar, Dakar BP 220, Sénégal

^2^ Ecole Supérieure Polytechnique, Université Cheikh Anta Diop de Dakar

^3^ Institute of Animal Hygiene and Veterinary Public Health University of Leipzig, Germany

^4^ Institute of Microbiology & Virology, Brandenburg Medical School Theodor Fontane, Germany

^5^ Animal Biology Department, Faculty of Sciences and Techniques, Cheikh Anta Diop University of Dakar, Dakar BP 5005, Senegal

**^**^Correspondance:** [idrissa.dieng@pasteur.sn](mailto:idrissa.dieng@pasteur.sn$)

^$^ These authors contributes equally

**KEYWORDS:** Chikungunya virus, CDC Trioplex RT-qPCR, Underperformance, West African Genotype, Pan Genotype, Detection improvement

**Table S1.** Description of CHIKV CDC trioplex assay and Alternative oligos (AltoDesign) sequences used during this study

| System |  | Séquences (5’-3’) | Target | Reporter - Quencher |
| --- | --- | --- | --- | --- |
| CHIKV CDC trioplex  Assay | Forward | ACCATCGGTGTTCCATCTAAAG | *nsp1* | **-** |
|  | Reverse | GCCTGGGCTCATCGTTATT | *nsp1* | **-** |
|  | Probe | ACAGTGGTTTCGTGTGAGGGCTAC | *nsp1* | **Hex - BHQ1** |
| CHIKV  AltoDesign  (This study) | Forward | ACC**H**TC**R**GTGTTCCATCTAAA**R** | *nsp1* | **-** |
|  | Reverse | GCC**Y**GGRCT**S**ATCGTTATT | *nsp1* | **-** |
|  | Probe | ACAGTGGTTTCGTG**Y**GARGGCTA**Y** | *nsp1* | **Hex - BHQ1** |

**Table S2.** Used CHIKV strains for assays comparison. The asterisk (*) highlighted the used WA genotype strain for analytical sensitivity tests

| ID Numbers | Genotype | CHIKV In House | Native Trioplex | AltoDesign |
| --- | --- | --- | --- | --- |
| SH431104 | WA | 18.06 | 28.16 | 18.01 |
| SH431105 | WA | 20.58 | 30.25 | 20.62 |
| SH431117 | WA | 18.43 | 29.38 | 19.25 |
| SH431164 | WA | 21.53 | 31.52 | 22.36 |
| SH431197 | WA | 20.63 | 31.06 | 20.73 |
| SH431204 | WA | 18 | 29.08 | 18.53 |
| SH431221 | WA | 24.4 | 33.9 | 25.15 |
| SH431229 | WA | 24.59 | 33.64 | 26.95 |
| PC CDC Trioplex Kit | Asian | 36.02 | 33.42 | 35.87 |
| CHIKV S27 | ECSA | 16 | 16.37 | 14.17 |
| SH274647 | WA | 18.17 | 29.46 | 20.02 |
| 3010/4 | WA | 14.15 | 20.11 | 12.91 |
| 3009/4 | WA | 14.06 | 21.67 | 13.31 |
| 2834/4 | WA | 13.79 | 21.85 | 12.55 |
| 2657/7 | WA | 15.73 | 23.03 | 14.36 |
| 16678/6 | WA | 18.26 | 19.6 | 22.9 |
| SH274640 | WA | 11.22 | 21.38 | 12.56 |
| ^**^SH274642 | WA | 11.76 | 22.39 | 13.41 |
| SH274646 | WA | 14.62 | 24.71 | 16.69 |
| SH274647 | WA | 18.09 | 26.8 | 19.97 |
| SH274648 | WA | 14.21 | 24.39 | 16.8 |
| HB78/4 | ECSA | 15.05 | 15.03 | 15.35 |
| A301 | ECSA | 14.32 | 16.53 | 15.06 |
| CS13 | ECSA | 16.66 | 19.35 | 17.41 |
| SH427924 | WA | 21.44 | 30.41 | 21.72 |
| SH427972 | WA | 29.46 | 34.04 | 29.63 |

**Table S3.** Average Cq values +/- standard deviation of Trioplex assays in comparison to AltoDesign using tenfold dilution

titred CHIKV WA genotype strain (SH274642).

| Amount in PFU per reaction | Trioplex (avg.Cq +/- S.D.) | AltoDesign (avg.Cq +/- S.D.) |
| --- | --- | --- |
| 32100000 | 25.34 ± 0.53 | 17.88 ± 0.12 |
| 3210000 | 28.6 ± 0.16 | 21.28 ± 0.15 |
| 321000 | 31.68 ± 0.07 | 25.14 ± 0.37 |
| 32100 | 34.65 ± 0.36 | 29.11 ± 0.07 |
| 3210 | NA | 32.56 ± 0.41 |
| 321 | NA | 34.97 ± 0.96 |
| 32.1 | NA | NA |

**Figure S1:** Comparison of Ct values for DENV (DENV 1-4) et ZIKV (African and Asian Lineages) determined by CDC Trioplex and Alto Design across tested strains.

^
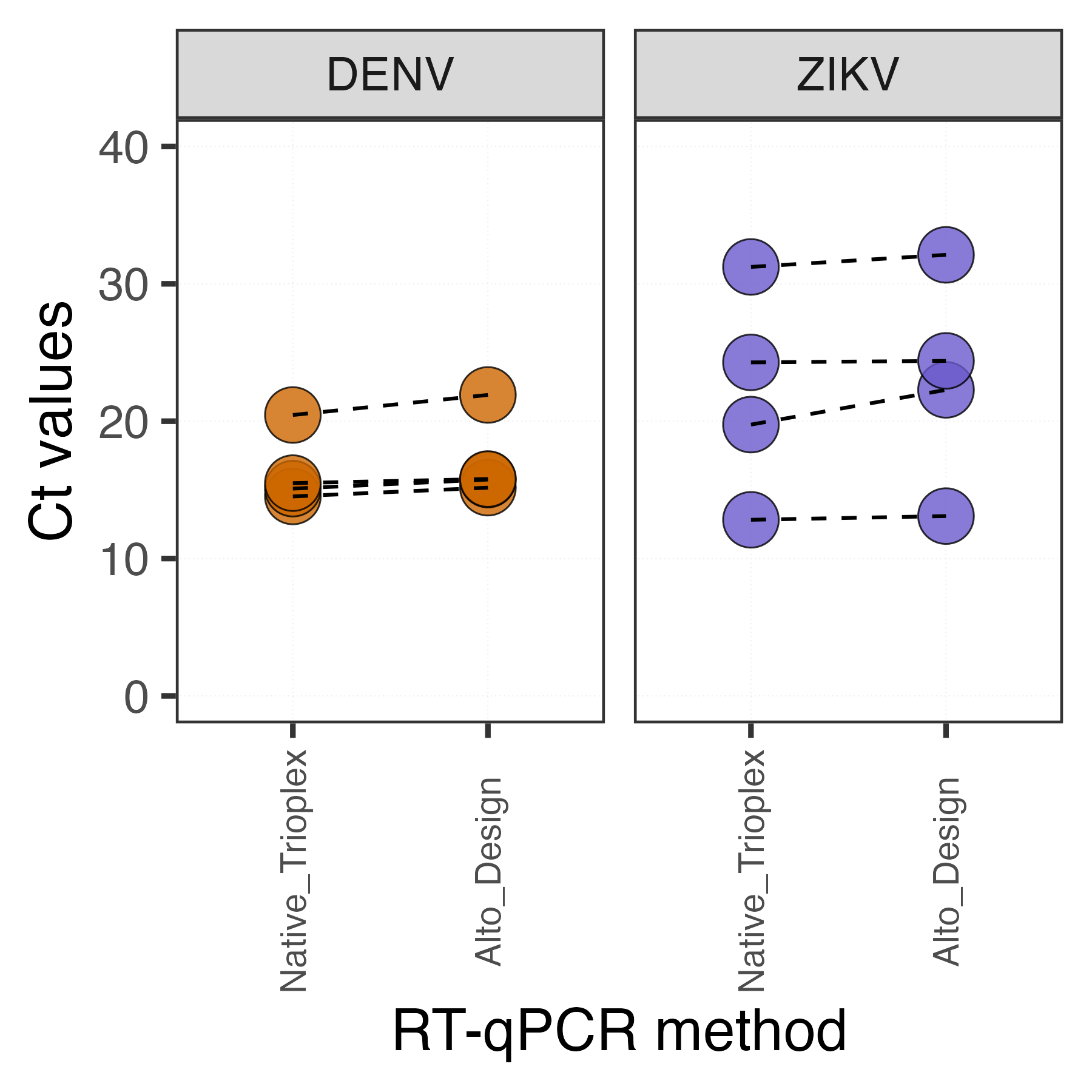
^
